# The Concise Guide to PHARMACOLOGY 2025/26: Nuclear hormone receptors

**DOI:** 10.1111/bph.70232

**Published:** 2025-12

**Authors:** Stephen P. H. Alexander, John A. Cidlowski, Alasdair J. Gibb, Eamonn Kelly, Alistair A. Mathie, Chloe J. Peach, Emma L. Veale, Jane F. Armstrong, Elena Faccenda, Simon D. Harding, Christopher Southan, Jamie A. Davies, Laurel Coons, Peter J. Fuller, Kenneth S. Korach, Donald P. McDonell, Robert Oakley, Sarah Radi, Rachid Safi, Frances Sladek, Morag J. Young

**Affiliations:** 1 Division of Physiology, Pharmacology & Neuroscience, School of Life Sciences, University of Nottingham Medical School, Nottingham, NG7 2UH, UK; 2 National Institute of Environmental Health Sciences, National Institutes of Health, Department of Health and Human Services, Research Triangle Park, NC 27709, USA; 3 Research Department of Neuroscience, Physiology and Pharmacology, Division of Biosciences, University College London, Gower Street, London, WC1E 6BT, UK; 4 School of Psychology and Neuroscience, University of Bristol, Bristol, BS8 1TD, UK; 5 School of Life Sciences, University of Westminster, London, W1W 6UW, UK; 6 Division of Physiology, Pharmacology & Neuroscience, School of Life Sciences, Centre of Membrane Proteins and Receptors (COMPARE), University of Nottingham Medical School, Nottingham, NG7 2UH, UK; 7 Medway School of Pharmacy, Universities of Kent and Greenwich, Chatham Maritime, Kent, ME4 4TB, UK; 8 Institute for Neuroscience and Cardiovascular Research, University of Edinburgh, Edinburgh, EH8 9XD, UK; 9 Duke University Medical Center, Durham, USA; 10 Hudson Institute of Medical Research, Clayton, Australia; 11 National Institutes of Health, Research Triangle Park, USA; 12 Duke University School of Medicine, Durham, USA; 13 University of California, Riverside, Riverside, USA; 14 Monash University, Melbourne, Australia

## Abstract

The Concise Guide to Pharmacology 2025/26 marks the seventh edition in this series of biennial publications in the British Journal of Pharmacology. Presented in landscape format, the guide provides a comparative overview of the pharmacology of drug target families. The concise nature of the Concise Guide refers to the style of presentation, being clear, accessible, and well-structured, rather than the scope of the content, which spans approximately 500 pages. The Concise Guide summarises the key pharmacological properties of around 1900 human drug targets, and nearly 7000 interactions, involving around 4400 ligands. While the content is a substantially condensed version of the more detailed information and links available at the www.guidetopharmacology.org website, the printed guide serves as a permanent, citable, point-in-time record, that remains stable despite ongoing updates to the online database. The full contents of this publication can be found at https://bpspubs.onlinelibrary.wiley.com/doi/10.1111/bph.70232.

The Concise Guides provide expert-curated recommendations of ‘Gold Standard’ selective pharmacological tools, available either commercially or as donations, which enable the identification of individual drug targets or families of drug targets. While the Concise Guide offers a more streamlined overview, more comprehensive information, including detailed pharmacological profiles and links to multiple online databases, is available through the Guide to Pharmacology website. The 2025/26 edition of the Concise Guide is based on material current as of mid-2025, and supersedes all previous editions, including the 2023/24 Guide, and earlier Guides to Receptors and Channels. It is produced in close conjunction with the Nomenclature and Standards Committee of the International Union of Basic and Clinical Pharmacology (NC-IUPHAR), and as such provides official IUPHAR classification and nomenclature for human drug targets, where applicable.

Nuclear hormone receptors are one of the six major pharmacological targets into which the Guide is divided, with the others being: G protein-coupled receptors, ion channels, catalytic receptors, enzymes and transporters. Each section includes nomenclature guidance, concise summaries information of the best available pharmacological tools, key references, and suggestions for further reading.

## Introduction:

Nuclear receptors are specialised transcription factors with commonalities of sequence and structure, which bind as homo- or heterodimers to specific consensus sequences of DNA (response elements) in the promoter region of particular target genes. They regulate (either promoting or repressing) transcription of these target genes in response to a variety of endogenous ligands. Endogenous agonists are hydrophobic entities which, when bound to the receptor promote conformational changes in the receptor to allow recruitment (or dissociation) of protein partners, generating a large multiprotein complex [23].

Two major subclasses of nuclear receptors with identified endogenous agonists can be identified: steroid and non-steroid hormone receptors. Steroid hormone receptors function typically as dimeric entities and are thought to be resident outside the nucleus in the unliganded state in a complex with chaperone proteins, which are liberated upon agonist binding. Migration to the nucleus and interaction with other regulators of gene transcription, including RNA polymerase, acetyltransferases and deacetylases, allows gene transcription to be regulated. Non-steroid hormone receptors typically exhibit a greater distribution in the nucleus in the unliganded state and interact with other nuclear receptors to form heterodimers, as well as with other regulators of gene transcription, leading to changes in gene transcription upon agonist binding.

Selectivity of gene regulation is brought about through interaction of nuclear receptors with particular consensus sequences of DNA, which are arranged typically as repeats or inverted palindromes to allow accumulation of multiple transcription factors in the promoter regions of genes.

## 1A. Thyroid hormone receptors

Nuclear hormone receptors → 1A. Thyroid hormone receptors

### Overview:

Thyroid hormone receptors (**TRs, nomenclature as agreed by the** **NC-IUPHAR** **Subcommittee on Nuclear Hormone Receptors** [1, 46]) are nuclear hormone receptors of the NR1A family, with diverse roles regulating macronutrient metabolism, cognition and cardiovascular homeostasis. TRs are activated by thyroxine (T_4_) and thyroid hormone (triiodothyronine). Once activated by a ligand, the receptor acts as a transcription factor either as a monomer, homodimer or heterodimer with members of the retinoid X receptor family. NH-3 has been described as an antagonist at TRs with modest selectivity for TRβ [121].

**Table:** 

Nomenclature	Thyroid hormone receptor-α	Thyroid hormone receptor-β
Systematic nomenclature	NR1A1	NR1A2
HGNC, UniProt	THRA, P10827	THRB, P10828
Rank order of potency	triiodothyronine > T_4_	triiodothyronine > T_4_
Agonists	dextrothyroxine [22]	dextrothyroxine [22]
Selective agonists	-	sobetirome [29, 147]

### Comments:

An interaction with integrin αVβ3 has been suggested to underlie plasma membrane localization of TRs and non-genomic signalling [11]. One splice variant, TRα_2_, lacks a functional DNA-binding domain and appears to act as a transcription suppressor.

Although radioligand binding assays have been described for these receptors, the radioligands are not commercially available.

## 1B. Retinoic acid receptors

Nuclear hormone receptors → 1B. Retinoic acid receptors

### Overview:

Retinoic acid receptors (**nomenclature as agreed by the** **NC-IUPHAR** **Subcommittee on Nuclear Hormone Receptors** [2, 53]) are nuclear hormone receptors of the NR1B family activated by the vitamin A-derived agonists tretinoin (ATRA) and alitretinoin, and the RAR-selective synthetic agonists TTNPB and adapalene. BMS493 is a family-selective antagonist [55].

**Table:** 

Nomenclature	Retinoic acid receptor-α	Retinoic acid receptor-β	Retinoic acid receptor-γ
Systematic nomenclature	NR1B1	NR1B2	NR1B3
HGNC, UniProt	RARA, P10276	RARB, P10826	RARG, P13631
Agonists	tretinoin [28]	tretinoin [28]	tretinoin [28]
Sub/family-selective agonists	tazarotene [28]	tazarotene [28], adapalene [27]	tazarotene [28], adapalene [27]
Selective agonists	BMS753 [52], tamibarotene [168], Ro 40-6055 [37]	AC261066 [100], AC55649 [99, 100]	AHPN [27]
Selective antagonists	Ro 41-5253 (pIC_50_ 6.3–7.2) [3, 78]	-	MM 11253 [87]

### Comments:

Ro 41-5253 has been suggested to be a PPARγ agonist [146]. LE135 is an antagonist with selectivity for RARα and RARβ compared with RARγ [96].

## 1C. Peroxisome proliferator-activated receptors

Nuclear hormone receptors → 1C. Peroxisome proliferator-activated receptors

### Overview:

Peroxisome proliferator-activated receptors (**PPARs, nomenclature as agreed by the** **NC-IUPHAR** **Subcommittee on Nuclear Hormone Receptors** [2, 112]) are nuclear hormone receptors of the NR1C family, with diverse roles regulating lipid homeostasis, cellular differentiation, proliferation and the immune response. PPARs have many potential endogenous agonists [16, 112], including 15-deoxy-Δ^12,14^-PGJ_2_, prostacyclin (PGI_2_), many fatty acids and their oxidation products, lysophosphatidic acid (LPA) [109], 13-HODE, 15S-HETE, Paz-PC, azelaoyl-PAF and leukotriene B4 (LTB_4_). Bezafibrate acts as a non-selective agonist for the PPAR family [177]. These receptors also bind hypolipidaemic drugs (PPARα) and anti-diabetic thiazolidinediones (PPARγ), as well as many non-steroidal anti-inflammatory drugs, such as sulindac and indomethacin. Once activated by a ligand, the receptor forms a heterodimer with members of the retinoid X receptor family and can act as a transcription factor. Although radioligand binding assays have been described for all three receptors, the radioligands are not commercially available. Commonly, receptor occupancy studies are conducted using fluorescent ligands and truncated forms of the receptor limited to the ligand binding domain.

**Table:** 

Nomenclature	Peroxisome proliferator-activated receptor-α	Peroxisome proliferator-activated receptor-β/δ	Peroxisome proliferator-activated receptor-γ
Systematic nomenclature	NR1C1	NR1C2	NR1C3
HGNC, UniProt	PPARA, Q07869	PPARD, Q03181	PPARG, P37231
Selective agonists	GW7647 [20, 21], CP-775146 [75], pirinixic acid [177], gemfibrozil [34]	GW0742X [58, 159], GW501516 [125]	GW1929 [20], bardoxolone (Partial agonist) [171], rosiglitazone [66, 92, 184], troglitazone [66, 184], pioglitazone [66, 144, 184], ciglitazone [66]
Selective antagonists	GW6471 (pIC_50_ 6.6) [181]	GSK0660 (pIC_50_ 6.5) [148]	T0070907 (p*K*_i_ 9) [88], GW9662 (Irreversible inhibition) (pIC_50_ 8.1) [90], CDDO-Me (p*K*_i_ 6.9) [171]

### Comments:

As with the estrogen receptor antagonists, many agents show tissue-selective efficacy (*e.g*. [15, 120, 141]). Agonists with mixed activity at PPARα and PPARγ have also been described (*e.g* [38, 62, 182]).

## 1D. Rev-Erb receptors

Nuclear hormone receptors → 1D. Rev-Erb receptors

### Overview:

Rev-erb receptors (**nomenclature as agreed by the** **NC-IUPHAR** **Subcommittee on Nuclear Hormone Receptors** [2, 10]) have yet to be officially paired with an endogenous ligand, but are thought to be activated by heme.

**Table:** 

Nomenclature	Rev-Erb-α	Rev-Erb-β
Systematic nomenclature	NR1D1	NR1D2
HGNC, UniProt	NR1D1, P20393	NR1D2, Q14995
Endogenous agonists	heme [138, 183]	heme [108, 138, 183]
Selective agonists	GSK4112 [60], GSK4112 [83]	-
Selective antagonists	SR8278 (pIC_50_ 6.5) [83]	-

## 1F. Retinoic acid-related orphans

Nuclear hormone receptors → 1F. Retinoic acid-related orphans

### Overview:

Retinoic acid receptor-related orphan receptors (**ROR, nomenclature as agreed by the** **NC-IUPHAR** **Subcommittee on Nuclear Hormone Receptors** [2, 10]) have yet to be assigned a definitive endogenous ligand, although RORα may be synthesized with a ‘captured’ agonist such as cholesterol [73, 74].

**Table:** 

Nomenclature	RAR-related orphan receptor-α	RAR-related orphan receptor-β	RAR-related orphan receptor-γ
Systematic nomenclature	NR1F1	NR1F2	NR1F3
HGNC, UniProt	RORA, P35398	RORB, Q92753	RORC, P51449
Endogenous agonists	cholesterol [74, 127]	-	-
Selective agonists	7-hydroxycholesterol [17], cholesterol sulphate [17, 74]	-	-
Comments	-	-	The immune system function of RORC proteins most likely resides with expression of the RORγt isoform by immature CD4^+^/CD8^+^ cells in the thymus [40, 158] and in lymphoid tissue inducer (LTi) cells [41].

### Comments:

Tretinoin shows selectivity for RORβ within the ROR family [154]. RORα has been suggested to be a nuclear receptor responding to melatonin [176].

## 1H. Liver X receptor-like receptors

Nuclear hormone receptors → 1H. Liver X receptor-like receptors

### Overview:

Liver X and farnesoid X receptors (LXR and FXR, **nomenclature as agreed by the** **NC-IUPHAR** **Subcommittee on Nuclear Hormone Receptors** [2, 116]) are members of a steroid analogue-activated nuclear receptor subfamily, which form heterodimers with members of the retinoid X receptor family. Endogenous ligands for LXRs include hydroxycholesterols (OHC), while FXRs appear to be activated by bile acids. In humans and primates, *NR1H5P* is a pseudogene. However, in other mammals, it encodes a functional nuclear hormone receptor that appears to be involved in cholesterol biosynthesis [126].

**Table:** 

Nomenclature	Farnesoid X receptor	Farnesoid X receptor-β	Liver X receptor-α	Liver X receptor-β
Systematic nomenclature	NR1H4	NR1H5	NR1H3	NR1H2
HGNC, UniProt	NR1H4, Q96RI1	NR1H5P, -	NR1H3, Q13133	NR1H2, P55055
Potency order	chenodeoxycholic acid > lithocholic acid, deoxycholic acid [103, 128]	-	20S-hydroxycholesterol, 22R-hydroxycholesterol, 24(S)-hydroxycholesterol >25-hydroxycholesterol, 27-hydroxycholesterol [70, 91]	20S-hydroxycholesterol,22R-hydroxycholesterol, 24(S)-hydroxycholesterol >25-hydroxycholesterol, 27-hydroxycholesterol [70, 91]
Endogenous agonists	-	lanosterol [126] - Mouse	-	-
Selective agonists	tropifexor [163], GW4064 [105], obeticholic acid [129], fexaramine [39]	-	-	BMS-852927 [80], GSK9772 [26], CE9A215 [7, 122]
Selective antagonists	DY268 (pIC_50_ 8.1) [186], guggul- sterone (pIC_50_ 5.7–6) [165, 179]	-	-	-

### Comments:

T0901317 [139] and GW3965 [30] are synthetic agonists that activate both LXRα and LXRβ with less than 10-fold selectivity between the two isoforms. In contrast, SR9243 [47] and BE1218 [180] are synthetic inverse agonists of both LXRα and LXRβ.

## 1I. Vitamin D receptor-like receptors

Nuclear hormone receptors → 1I. Vitamin D receptor-like receptors

### Overview:

Vitamin D (VDR), Pregnane X (PXR) and Constitutive Androstane (CAR) receptors (**nomenclature as agreed by the** **NC-IUPHAR** **Subcommittee on Nuclear Hormone Receptors** [2, 116]) are members of the NR1I family of nuclear receptors, which form heterodimers with members of the retinoid X receptor family. PXR and CAR are activated by a range of exogenous compounds, with no established endogenous physiological agonists, although high concentrations of bile acids and bile pigments activate PXR and CAR [116].

**Table:** 

Nomenclature	Vitamin D receptor	Pregnane X receptor	Constitutive androstane receptor
Systematic nomenclature	NR1I1	NR1I2	NR1I3
HGNC, UniProt	VDR, P11473	NR1I2, O75469	NR1I3, Q14994
Endogenous agonists	1,25-dihydroxyvitamin D3 [14, 44]	17β-estradiol [72]	-
Selective agonists	seocalcitol [31, 175], doxercalciferol	hyperforin [117, 174], 5β-pregnane-3,20-dione [72], lovastatin [93], rifampicin [18, 93]	TCPOBOP [164] - Mouse, CITCO [102]
Selective antagonists	TEI-9647 (pIC_50_ 8.2) [143] - Chicken, ZK159222 (pIC_50_ 7.5) [48, 67]	-	-
Comments	-	-	Clotrimazole [118] and T0901317 [76] although acting at other sites, function as antagonists of the constitutive androstane receptor.

## 2A. Hepatocyte nuclear factor-4 receptors

Nuclear hormone receptors → 2A. Hepatocyte nuclear factor-4 receptors

### Overview:

The nomenclature of hepatocyte nuclear factor-4 receptors is agreed by the **NC-IUPHAR** **Subcommittee on Nuclear Hormone Receptors** [2, 10]. While linoleic acid has been identified as the endogenous ligand for HNF4α its function remains ambiguous [187]. HNF4γ has yet to be paired with an endogenous ligand.

**Table:** 

Nomenclature	Hepatocyte nuclear factor-4-α	Hepatocyte nuclear factor-4-γ
Systematic nomenclature	NR2A1	NR2A2
HGNC, UniProt	HNF4A, P41235	HNF4G, Q14541
Endogenous agonists	linoleic acid [187]	-
Agonists	alverine [89], benfluorex [89]	-
Selective agonists	HNF4α inhibitor	-
Selective antagonists	BI6015 [81]	-
Comments	HNF4α was purified originally from liver but is also found in other tissues including the kidney, intestines, pancreas and stomach [137, 151]. Its expression is driven by two promoters, leading to several isoform variants, the abundance of which vary between the tissues where HNF4 is expressed [82]. HNF4α has constitutive transactivation activity [187] and binds DNA as a homodimer [71].	HNF4γ is structurally very similar to HNF4α although its expression is restricted to the intestinal tract. HNF4γ has two known isoforms, though it is unknown if this is due to alternative promoter usage or alternative splicing. The two HNF4 receptors can bind DNA as heterodimers as well as homodimers [167].

## 2B. Retinoid X receptors

Nuclear hormone receptors → 2B. Retinoid X receptors

### Overview:

Retinoid X receptors (**nomenclature as agreed by the** **NC-IUPHAR** **Subcommittee on Nuclear Hormone Receptors** [2, 54]) are NR2B family members activated by alitretinoin and the RXR-selective agonists bexarotene and LG100268, sometimes referred to as rexinoids. UVI3003 [119] and HX 531 [42] have been described as a pan-RXR antagonists. These receptors form RXR-RAR heterodimers and RXR-RXR homodimers [25, 107].

**Table:** 

Nomenclature	Retinoid X receptor-α	Retinoid X receptor-β	Retinoid X receptor-γ
Systematic nomenclature	NR2B1	NR2B2	NR2B3
HGNC, UniProt	RXRA, P19793	RXRB, P28702	RXRG, P48443
Sub/family-selective agonists	bexarotene [19, 24, 160]	bexarotene [19, 24, 160]	bexarotene [19, 24, 160]
Selective agonists	CD3254 [56]	-	-

## 2C. Testicular receptors

Nuclear hormone receptors → 2C. Testicular receptors

### Overview:

Testicular receptors **(nomenclature as agreed by the** **NC-IUPHAR** **Subcommittee on Nuclear Hormone Receptors** [10]) have yet to be officially paired with an endogenous ligand, although testicular receptor 4 has been reported to respond to retinoids.

**Table:** 

Nomenclature	Testicular receptor 2	Testicular receptor 4
Systematic nomenclature	NR2C1	NR2C2
HGNC, UniProt	NR2C1, P13056	NR2C2, P49116
Endogenous agonists	-	retinol [193], tretinoin [193]
Comments	Forms a heterodimer with TR4; gene disruption appears without effect on testicular development or function [149].	Forms a heterodimer with TR2.

## 2E. Tailless-like receptors

Nuclear hormone receptors → 2E. Tailless-like receptors

### Overview:

Tailless-like receptors (**nomenclature as agreed by the** **NC-IUPHAR** **Subcommittee on Nuclear Hormone Receptors** [10]) have yet to be officially paired with an endogenous ligand.

**Table:** 

Nomenclature	TLX	PNR
Systematic nomenclature	NR2E1	NR2E3
HGNC, UniProt	NR2E1, Q9Y466	NR2E3, Q9Y5X4
Agonists	BMS493 [61], tretinoin [61]	-
Comments	Gene disruption is associated with abnormal brain development [86, 115].	-

## 2F. COUP-TF-like receptors

Nuclear hormone receptors → 2F. COUP-TF-like receptors

### Overview:

COUP-TF-like receptors (**nomenclature as agreed by the** **NC-IUPHAR** **Subcommittee on Nuclear Hormone Receptors** [2, 10]) have yet to be officially paired with an endogenous ligand.

**Table:** 

Nomenclature	COUP-TF1	COUP-TF2	V-erbA-related gene
Systematic nomenclature	NR2F1	NR2F2	NR2F6
HGNC, UniProt	NR2F1, P10589	NR2F2, P24468	NR2F6, P10588
Comments	Gene disruption is perinatally lethal [136].	Gene disruption is embryonically lethal [130].	Gene disruption impairs CNS development [173].

## 3B. Estrogen-related receptors

Nuclear hormone receptors → 3B. Estrogen-related receptors

### Overview:

Estrogen-related receptors (**nomenclature as agreed by the** **NC-IUPHAR** **Subcommittee on Nuclear Hormone Receptors** [10]) have yet to be officially paired with an endogenous ligand.

**Table:** 

Nomenclature	Estrogen-related receptor-α	Estrogen-related receptor-β	Estrogen-related receptor-γ
Systematic nomenclature	NR3B1	NR3B2	NR3B3
HGNC, UniProt	ESRRA, P11474	ESRRB, O95718	ESRRG, P62508
Comments	Activated by some dietary flavonoids [155]; activated by the synthetic agonist GSK4716 [196] and blocked by XCT790 [178].	May be activated by DY131 [185].	May be activated by DY131 [185].

## 4A. Nerve growth factor IB-like receptors

Nuclear hormone receptors → 4A. Nerve growth factor IB-like receptors

### Overview:

Nerve growth factor IB-like receptors (**nomenclature as agreed by the**NC-IUPHAR Subcommittee on Nuclear Hormone Receptors [10]) have yet to be officially paired with an endogenous ligand, although preliminary evidence suggests that they bind fatty acid metabolites and fatty acid mimetic drugs [6, 35, 133].

**Table:** 

Nomenclature	Nerve Growth factor IB	Nuclear receptor related 1	Neuron-derived orphan receptor 1
Systematic nomenclature	NR4A1	NR4A2	NR4A3
HGNC, UniProt	NR4A1, P22736	NR4A2, P43354	NR4A3, Q92570
Comments	An endogenous agonist, cytosporone B, has been described [188], although structural analysis and molecular modelling has not identified a ligand binding site [5, 45, 172].	-	-

## 5A. Fushi tarazu F1-like receptors

Nuclear hormone receptors → 5A. Fushi tarazu F1-like receptors

### Overview:

Fushi tarazu F1-like receptors (**nomenclature as agreed by the** **NC-IUPHAR** **Subcommittee on Nuclear Hormone Receptors** [10]) have yet to be officially paired with an endogenous ligand.

**Table:** 

Nomenclature	Steroidogenic factor 1	Liver receptor homolog-1
Systematic nomenclature	NR5A1	NR5A2
HGNC, UniProt	NR5A1, Q13285	NR5A2, O00482
Comments	Reported to be inhibited by AC45594 [36] and SID7969543 [101].	-

## 6A. Germ cell nuclear factor receptors

Nuclear hormone receptors → 6A. Germ cell nuclear factor receptors

### Overview:

Germ cell nuclear factor receptors (**nomenclature as agreed by the** **NC-IUPHAR** **Subcommittee on Nuclear Hormone Receptors** [10]) have yet to be officially paired with an endogenous ligand.

**Table:** 

Nomenclature	Germ cell nuclear factor
Systematic nomenclature	NR6A1
HGNC, UniProt	NR6A1, Q15406

## 0B. DAX-like receptors

Nuclear hormone receptors → 0B. DAX-like receptors

### Overview:

Dax-like receptors (**nomenclature as agreed by the** **NC-IUPHAR** **Subcommittee on Nuclear Hormone Receptors** [10]) have yet to be officially paired with an endogenous ligand.

**Table:** 

Nomenclature	DAX1	SHP
Systematic nomenclature	NR0B1	NR0B2
HGNC, UniProt	NR0B1, P51843	NR0B2, Q15466

## Steroid hormone receptors

Nuclear hormone receptors → Steroid hormone receptors

### Overview:

Steroid hormone receptors (**nomenclature as agreed by the** **NC-IUPHAR** **Subcommittee on Nuclear Hormone Receptors** [2, 33, 98]) are nuclear hormone receptors of the NR3 class, with endogenous agonists that may be divided into 3-hydroxysteroids (estrone and 17β-estradiol) and 3-ketosteroids (dihydrotestosterone [DHT], aldosterone, cortisol, corticosterone, progesterone and testosterone). These receptors exist as dimers coupled with chaperone molecules (such as hsp90β (*HSP90AB1*, P08238) and immunophilin FKBP52:*FKBP4*, Q02790), which are shed on binding the steroid hormone. Although rapid signalling phenomena are observed [95, 135], the principal signalling cascade appears to involve binding of the activated receptors to nuclear hormone response elements of the genome, with a 15-nucleotide consensus sequence AGAACAnnnTGTTCT (*i.e.* an inverted palindrome) as homo- or heterodimers. They also affect transcription by protein-protein interactions with other transcription factors, such as activator protein 1 (AP-1) and nuclear factor κB (NF-κB). Splice variants of each of these receptors can form functional or non-functional monomers that can dimerize to form functional or non-functional receptors. For example, alternative splicing of PR mRNA produces A and B monomers that combine to produce functional AA, AB and BB receptors with distinct characteristics [166].

A 7TM receptor responsive to estrogen (*GPER1*, Q99527, also known as GPR30, see [134]) has been described. Human orthologues of 7TM ’membrane progestin receptors’ (*PAQR7*, *PAQR8* and *PAQR5*), initially discovered in fish [194, 195], appear to localize to intracellular membranes and respond to ’non-genomic’ progesterone analogues independently of G proteins [152].

## 3A. Estrogen receptors

Nuclear hormone receptors → Steroid hormone receptors → 3A. Estrogen receptors

### Overview:

Estrogen receptor (ER) activity regulates diverse physiological processes *via* transcriptional modulation of target genes [2]. The selection of target genes and the magnitude of the response, be it induction or repression, are determined by many factors, including the effect of the hormone ligand and DNA binding on ER structural conformation, and the local cellular regulatory environment. The cellular environment defines the specific complement of DNA enhancer and promoter elements present and the availability of coregulators to form functional transcription complexes. Together, these determinants control the resulting biological response.

**Table:** 

Nomenclature	Estrogen receptor-α	Estrogen receptor-β
Systematic nomenclature	NR3A1	NR3A2
HGNC, UniProt	ESR1, P03372	ESR2, Q92731
Endogenous agonists	estriol [85], estrone [85]	-
Selective agonists	propylpyrazoletriol [84, 153], ethinylestradiol [69]	WAY200070 [104], diarylpropionitrile [111, 153], prinaberel [32, 104]
Sub/family-selective antagonists	bazedoxifene (pIC_50_ 7.6) [114]	bazedoxifene (pIC_50_ 7.1) [114]
Selective antagonists	clomiphene (p*K*_i_ 8.9) [4], methyl-piperidino-pyrazole (p*K*_i_ 8.6) [156]	R,R-THC (p*K*_i_ 8.4) [110, 157], PHTPP (p*K*_i_ 6.9) [192]

### Comments:

R,R-THC exhibits partial agonist activity at ERα [110, 157]. Estrogen receptors may be blocked non-selectively by tamoxifen and raloxifene and labelled by [^3^H]17β-estradiol and [^3^H]tamoxifen. Many agents thought initially to be antagonists at estrogen receptors appear to have tissue-specific efficacy (*e.g*. Tamoxifen is an antagonist at estrogen receptors in the breast, but is an agonist at estrogen receptors in the uterus), hence the descriptor SERM (selective estrogen receptor modulator) or SnuRM (selective nuclear receptor modulator). Y134 has been suggested to be an ERα-selective estrogen receptor modulator [123].

## 3C. 3-Ketosteroid receptors

Nuclear hormone receptors → Steroid hormone receptors → 3C. 3-Ketosteroid receptors

### Overview:

Steroid hormone receptors (**nomenclature as agreed by the** **NC-IUPHAR** **Subcommittee on Nuclear Hormone Receptors** [2, 33, 98]) are nuclear hormone receptors of the NR3 class, with endogenous agonists that may be divided into 3-hydroxysteroids (estrone and 17β-estradiol) and 3-ketosteroids (dihydrotestosterone [DHT], aldosterone, cortisol, corticosterone, progesterone and testosterone). For rodent GR and MR, the physiological ligand is corticosterone rather than cortisol. Clinically-used drugs interacting with the 3-ketosteroid receptors include testosterone (AR), methylprednisolone (GR), eplerenone (MR), and medroxyprogesterone (PR).

**Table:** 

Nomenclature	Androgen receptor	Glucocorticoid receptor
Systematic nomenclature	NR3C4	NR3C1
HGNC, UniProt	AR, P10275	NR3C1, P04150
Rank order of potency	dihydrotestosterone > testosterone	cortisol, corticosterone» aldosterone, deoxycortisone [142]
Endogenous agonists	dihydrotestosterone [161]	-
Selective agonists	testosterone propionate [106], mibolerone [57], fluoxymesterone [68], methyltrienolone [170], dromostanolone propionate	fluticasone propionate [13], flunisolide [4], beclometasone [4], methylprednisolone [4], betamethasone [4], budesonide [113]
Selective antagonists	bicalutamide (pK_i_ 7.7) [79], PF0998425 (pIC_50_ 7.1–7.5) [97], enzalutamide (pIC_50_ 7.4) [162], nilutamide (pIC_50_ 7.1–7.1) [150], hydroxyflutamide (pEC_50_ 6.6) [170], galeterone (pIC_50_ 6.4) [64], flutamide (Displacement of ^3^[H] testosterone from wild-type androgen receptors) (p*K*_i_ 5.4) [169]	onapristone (pIC_50_ 7.6) [189], ZK112993
Labelled ligands	[^3^H]dihydrotestosterone (Selective Agonist),[^3^H]methyltrienolone (Selective Agonist), [^3^H]mibolerone (Agonist)	[^3^H]dexamethasone (Agonist)

**Table:** 

Nomenclature	Mineralocorticoid receptor	Progesterone receptor
Systematic nomenclature	NR3C2	NR3C3
HGNC, UniProt	NR3C2, P08235	PGR, P06401
Rank order of potency	corticosterone, cortisol, aldosterone, progesterone [142]	progesterone
Endogenous agonists	deoxycorticosterone [142], aldosterone [65, 142], cortisol [65, 142], corticosterone	progesterone [43]
Selective agonists	fludrocortisone [142]	medroxyprogesterone (Affinity at human PR-A) [190], ORG2058, levonorgestrel [12, 145]
Endogenous antagonists	progesterone (pIC_50_ 11) [142]	-
Antagonists	spironolactone (p*K*_i_ 8.6) [9], eplerenone (pIC_50_ 6.4), canrenone, drospirenone [132]	-
Selective antagonists	esaxerenone (pIC_50_ 8.6) [77, 131], finerenone (pIC_50_ 7.7) [8], balcinrenone (pIC_50_ 6.4) [59], onapristone (pIC_50_ 6.3) [189]	ulipristal acetate (pIC_50_ 9.7) [140], mifepristone (Mixed) (p*K*_i_ 9) [191], onapristone (p*K*_i_ 7.7) [63], ZK112993
Labelled ligands	[^3^H] corticosterone (Agonist) [124],[^3^H] cortisol (Agonist) [124],[^3^H]aldosterone (Agonist) [49]	[^3^H]ORG2058 (Selective Agonist)
Comments	Pre-receptor ligand specificity is provided for the MR in tissues associated with maintenance of sodium homeostasis by the co-expression of 11β-hydroxysteroid dehydrogenase type II which converts cortisol (corticosterone in rodents) to their inactive forms. Given the increasing use of Danio rerio (zebrafish) as an experimental model, it is important to note that progesterone (and spironolactone) is a MR agonist in fish [50]. For GR and MR in rodents, the physiological ligand is corticosterone rather than cortisol.	-

### Comments:

[^3^H]dexamethasone also binds to MR*in vitro*. PR antagonists have been suggested to subdivide into Type I (*e.g.* onapristone) and Type II (*e.g*. ZK112993) groups. These groups appear to promote binding of PR to DNA with different efficacies and evoke distinct conformational changes in the receptor, leading to a transcription-neutral complex [51, 94]. Mutations in AR underlie testicular feminization and androgen insensitivity syndromes, spinal and bulbar muscular atrophy (Kennedy’s disease).
